# Robust flexural performance and fracture behavior of TiO_2_ decorated densified bamboo as sustainable structural materials

**DOI:** 10.1038/s41467-023-36939-6

**Published:** 2023-03-04

**Authors:** Ziyu Ba, Hongyun Luo, Juan Guan, Jun Luo, Jiajia Gao, Sujun Wu, Robert O. Ritchie

**Affiliations:** 1 School of Materials Science and Engineering, Beihang University, Beijing, P. R. China; 2 Beijing Advanced Innovation Centre for Biomedical Engineering, Beihang University, Beijing, P. R. China; 3 Beijing Key Laboratory of Advanced Nuclear Materials and Physics, Beihang University, Beijing, P. R. China; 4 Department of Materials Science & Engineering, University of California, Berkeley, CA 94720 USA

**Keywords:** Mechanical properties, Biomaterials, Design, synthesis and processing

## Abstract

High-performance, fast-growing natural materials with sustainable and functional features currently arouse significant attention. Here, facile processing, involving delignification, in situ hydrothermal synthesis of TiO_2_ and pressure densification, is employed to transform natural bamboo into a high-performance structural material. The resulting TiO_2_-decorated densified bamboo exhibits high flexural strength and elastic stiffness, with both properties more than double that of natural bamboo. Real-time acoustic emission reveals the key role of the TiO_2_ nanoparticles in enhancing the flexural properties. The introduction of nanoscale TiO_2_ is found to markedly increase the degree of oxidation and the formation of hydrogen bonds in bamboo materials, leading to extensive interfacial failure between the microfibers, a micro-fibrillation process that results in substantial energy consumption and high fracture resistance. This work furthers the strategy of the synthetic reinforcement of fast-growing natural materials, which could lead to the expanded applications of sustainable materials for high-performance structural applications.

## Abstract (web summary)

Fast-growing bamboo still need advanced processing before being fabricated into sustainable structural materials. Here, the authors develop high-performance TiO_2_ reinforced densified bamboo via in situ hydrothermal synthesis and reveal the flexural failure mechanism of the composite.

## Introduction

Biomass composites from animals and plants are increasingly being utilized for the sustainable development of society^1^ due to their renewability, high production, and often good mechanical properties^2,3^. For example, cellulose-based materials can be chemically extracted from materials, such as wood^4^, bamboo^5,6^, ramie plant^7^ and cotton^8^, with a high surface area and unique morphology; these natural materials display a low density and can achieve a high mechanical strength^9^, which gives them a variety of useful functionalities. Indeed, we see a rapid expansion of wood/cellulose-based nanotechnology^10^. To date, the “top-down” approach^11^ to manufacture high-performance cellulose materials includes delignification of wood or chemical treatment to partially remove lignin and hemicellulose to acquire bulk cellulose scaffolds^12,13^, densification through hot pressing^14,15^, vacuum impregnation of resin^16^ or mineral substances^17,18^, freeze-drying^19^, carbonization^20,21^. The impregnation of nanoparticles in wood has usually been enacted for functionalities such as fire retardance^17^, magnetic properties^18^, and transparency^22^, whereas the resulting mechanical performance has rarely been examined. Furthermore, most reports on the addition of nanoparticles have focused on the processing of wood, which as a xylophyta grows much slower than herbage such as bamboo.

Constituted by parenchymal cells and vascular bundles, bamboo is a fast-growing natural composite material with a high strength-to-weight ratio and unique natural hierarchical structure^5,23^. Highly oriented cellulose fibrils in bamboo cells provide stiffness, whereas the hemicellulose cell walls form the interfaces^24,25^. However, the uneven distribution of fibers, which can lead to the asymmetric flexural behavior of bamboo, can limit its future application^26^. Recently, Li et al.^27^ demonstrated that delignified bamboo could be fabricated into a tough structural material with a uniform fiber distribution by hot pressing; using this technique, the tensile strength of bamboo can be raised to ~1 GPa with a flexural strength as high as 553 MPa. Since the deformation and fracture mechanisms, and hence the resulting toughness, of such modified natural bamboo have never been investigated, the intent of the current work is to examine these aspects in order to seek a deeper understanding of the mechanical performance of densified bamboo.

TiO_2_ is a non-hazardous compound with excellent properties for photocatalysis, antifungal use, self-cleaning and hydrogen production, which has been a popular nano-reinforcement in composites^28–30^. Hydrothermal reactions permit the synthesis of TiO_2_ in substrates of wood, bamboo, and cottons with high porosity and water-swelling behavior^31^. In turn, when introducing TiO_2_ into bamboo, the photo-catalytic activity of the TiO_2_ nanoparticles can contribute to the effective antimicrobial property of the bamboo. In addition, the hydroxyl groups on the surface of delignified bamboo provide synthesis sites for TiO_2_^32^. Although a number of studies have sought to investigate the possibility of reinforcing giant reed with graphene oxide (GO) and SiC^33^, the in situ synthesis of nanoparticles, such as TiO_2_, in bamboo with its mechanical variations have rarely been mentioned. In the current study, a strategy for nanoparticle-reinforced delignified and densified bamboo was developed to induce a series of reinforcement mechanisms to markedly enhance properties.

One remarkable feature of natural bamboo is the high anisotropy of its morphology and fracture behavior; in particular, the toughness of natural bamboo can reach ten times of that of a fibrous composite with the same fraction of fibers^34^. Various damage mechanisms, especially fiber pull-out, contribute to this high toughness. Such processes can be readily identified by their resulting acoustic signals, which can be assessed using acoustic emission (AE) measurement. Indeed, the technique of AE has been extensively applied for real-time detection of fracture in many metals and composites by analyzing the amplitude, peak frequency, rise time and counts^35,36^. Previous research suggests that the AE signals during the deformation of bamboo can be associated with the characteristic damage modes, i.e. the fiber fractures generate high-frequency signals, whereas the fracture of the parenchymal cell/matrix generates low-frequency signals^37,38^. It was therefore reasoned that AE could aid the identification of the salient damage and fracture mechanisms in nanoparticle-reinforced densified bamboo.

Here, we show the fabrication of a composite bamboo by introducing nano-sized TiO_2_ particles using a facile hydrothermal synthesis into delignified and densified bamboo. Following characterization of the morphology and chemical properties, we conduct real-time fracture analysis using AE to reveal the various deformation modes and dominant toughening mechanisms in densified and micro-reinforced bamboo. By characterizing the multiple modes of fracture, our objective is to provide guidelines for the design and processing of reinforced natural bamboo materials to inspire their wider application as structural materials.

## Results

### Morphology and physio-chemical properties of TiO_2_-reinforced densified bamboo

**Fig. 1:**
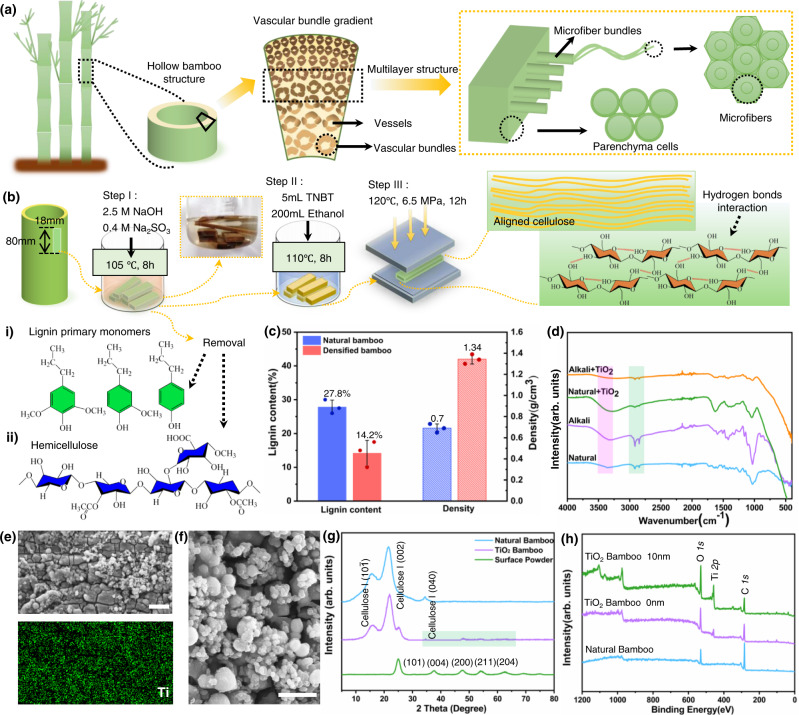
The preparation and characterization of densified TiO_2_ bamboo. **a** Multilevel structure of bamboo. **b** The three-step processes including delignification, synthesis of TiO_2_ and hot pressing. TNBT is the abbreviation of tetrabutyl titanate. (i) Lignin primary monomers, (ii) hemicellulose. **c** Lignin content and density of natural bamboo and densified bamboo. Data are presented as mean values ± SEM, *n* = 3 independent samples. **d** FTIR spectra of natural, alkali-treated and TiO_2_ decorated bamboo. The highlighted region, pink: the hydrogen groups, green: the methyl groups. **e** SEM and Energy-dispersive spectroscopy (EDS) images of TiO_2_ distribution on inner natural bamboo, inset EDS with color green: titanium. Scale bar is 10 μm. **f** High-magnification of SEM image, scale bar is 5 μm. **g** XRD patterns of natural bamboo, TiO_2_-decorated bamboo. The highlighted region in green: TiO_2_ on bamboo. **h** XPS spectra of natural bamboo and TiO_2_-decorated bamboo.

Bamboo is a multiscale microstructure fibrous composite; its cross-sectional structure is demonstrated in Fig. 1a. The vascular bundles are embedded in parenchyma cells, consist of microfiber bundles which aggregated microfibers which dimensions of ~15–20 μm. Cuboid bamboo specimens were prepared with the longest side along the direction of the growth of mature bamboo. The three-step processing involved the removal of lignin and hemicellulose by boiling in NaOH and Na_2_SO_3_ solutions, vacuum impregnation and hydrothermal synthesis of TiO_2_ in a reaction bath, and then densification by hot pressing (Fig. 1b and Supplementary Fig. 1). With such processing, the density of the bamboo material was increased by ~91% from 0.7 to 1.34 g/cm^3^ (Fig. 1c). After the alkali treatment, the peaks at 1423 cm^−1^ and 1510 cm^−1^ in the Fourier transform infrared (FTIR) spectra (characteristic of stretching vibrations of the benzene ring in lignin) and the peaks at 1850–1600 cm^−1^ (characteristic of stretching vibrations of the carboxyl group in xylan in hemicellulose) were decreased (Fig. 1d); this indicates the removal of lignin and hemicellulose from the bamboo^39,40^. Such chemical delignification caused the oxygen/carbon (O/C) ratio to increase from 0.23 to 0.60 (Table 1). X-ray diffraction (XRD) analysis also showed that all the characteristic peaks for the crystalline phase of cellulose-I, (101), (002) and (040), were present in the TiO_2_-modified densified bamboo. The enlarged XRD peaks at 2θ = 25.3°, 37.8°, and 48.0° (Fig. 1g), corresponding to the (101), (004), and (200) planes of anatase TiO_2_ (JCPDS 21-1272), served to confirm the successful synthesis of the TiO_2_. In addition, from the scanning electron microscopy (SEM) in Fig. 1e, f and Supplementary Fig. 2 and X-ray photoelectron spectroscopy (XPS) in Fig. 1h, the TiO_2_ particles can be seen to have been successfully introduced into the bamboo.

**Table 1:** Atomic composition (%) and peak deconvolution results of the C *1* *s* peak (the corresponding bonds) obtained by XPS analysis

	C	O	Ti	O/C	C1 (C–C, C–H)	C2 (C–O–C/C–OH)	C3 (O–C–O/C = O)	C_ox_/C_unox_
Natural	81.27%	18.73%	0	0.23	83.75%	14.43%	1.82%	0.19
TiO_2_-Reinforced	60.3%	35.91%	3.79%	0.60	57.23%	39.73%	3.04%	0.75

**Fig. 2:**
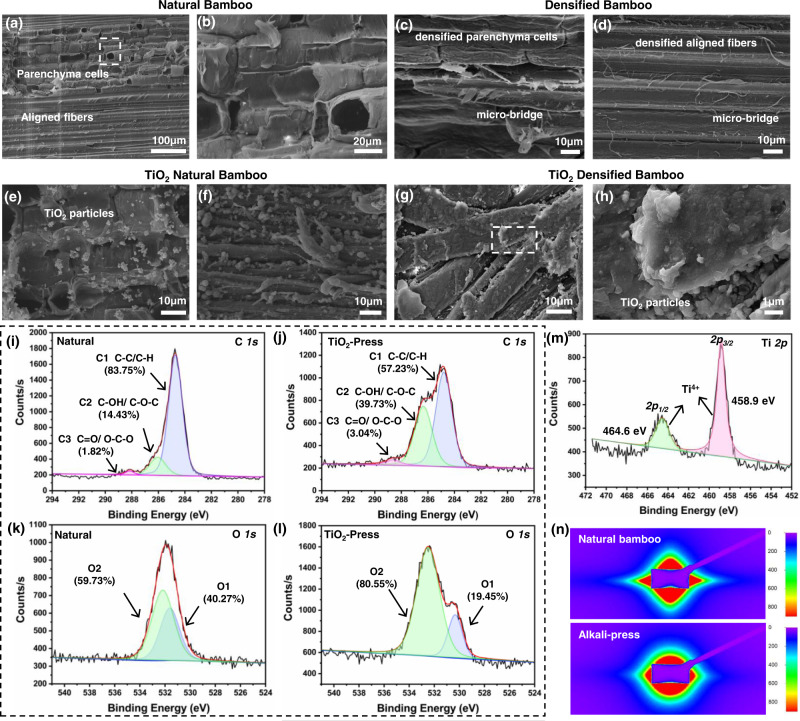
Morphology and chemical group composition of the bamboo materials. SEM images of (**a**, **b**) natural bamboo, white dashed line in (**a**) shows the SEM magnification region of (**b**). **c** Densified parenchyma cells in densified bamboo. **d** Densified aligned fibers. **e**, **f** TiO_2_-decorated bamboo, TiO_2_ particles on (**e**) parenchyma cells and (**f**) bamboo fibers. **g**, **h** TiO_2_ densified bamboo, white dashed line in (**g**) shows the SEM magnification region of (**h**). **i**, **j** High-resolution XPS spectra of C *1* *s* peaks at 282-290 eV of (**i**) natural bamboo, **j** densified TiO_2_ bamboo. **k**, **l** High-resolution XPS spectra of O *1* *s* peaks of (**k**) natural bamboo, (**l**) densified TiO_2_ bamboo. **m** High-resolution XPS spectra of Ti *2p* peaks of densified TiO_2_-decorated bamboo. **n** Small-angle X-ray scattering (SAXS) patterns of natural bamboo and densified bamboo.

The detailed morphologies of natural, densified and TiO_2_-modified bamboo are compared in Fig. 2a–h. In natural bamboo, parenchymal cells with numerous micrometer- and nanometer-sized voids together with bamboo microfiber bundles (aggregation of microfibers between vascular bundles) can be seen to be aligned adjacent to each other (Fig. 2a, b), the voids allowing the easy vacuum impregnation of fluids or reaction solutions. The delignification process removed most of the lignin and hemicellulose in the bamboo. As shown in the computed micro-tomography in Supplementary Fig. 3, ~190-μm-sized voids were generated between the vessels when the parenchyma cells were broken after the alkali treatment. Such voids can be compressed easily to produce denser bamboo materials using hot pressing. Compared to natural bamboo, the parenchyma cells are highly compact in the densified bamboo (Fig. 2c, d and Supplementary Fig. 3). The bamboo microfibers (single fiber of vascular bundles with dimensions of ~15–20 μm) become slightly twisted following the hot alkali treatment, but still remain highly aligned at the macroscale. This causes the 2D small-angle X-ray scattering (SAXS) patterns, shown in Fig. 2n and Supplementary Fig. 4, to transition from a rhombus shape in natural bamboo into a spindle shape after the treatment. These scattering patterns indicate the unchanged orientation of parallel cellulose fibers in the equator direction after the removal of lignin and hemicellulose and after the densification, similar to that reported in ref. ^41^.

Figure 2e shows that both the cell walls and vascular fibers of natural bamboo become decorated with TiO_2_ particles after the hydrothermal reaction; some aggregates of TiO_2_ particles, with dimensions ~500 nm to 1 μm, also appear at the interfaces of fibers of TiO_2_-densified bamboo, as shown in Fig. 2g, h and Supplementary Fig. 5. The thickness of the TiO_2_-densified bamboo was ~1.2–1.5 mm (Supplementary Fig. 6), with TiO_2_ nanoparticles detected at ~0.5 mm beneath the surface but not in the middle regions, probably because the reaction solution was not able to infiltrate completely into the internal bamboo cell walls. In addition, the bamboo matrix was also seen to form cracks due to the swelling stresses resulting from the high temperature of 120 °C in hot-pressing process.

To further reveal the differences in the chemical composition, peak deconvolution analysis was performed on the C and O peaks of the high-resolution XPS spectrum of natural and TiO_2_-modified bamboo (Fig. 2i–l). The C *1* *s* peak of the XPS spectra was mainly deconvoluted into three peaks (C1, C2, and C3) and the O *1* *s* peak into two peaks^42,43^ (O1 and O2), with each peak associated with a different chemical bond (as detailed in Table 1). After delignification and densification, the C1 peak decreased from 84 to 57% and O1 decreased from 40 to 19%, which is related to the removal of hydrocarbons, i.e., lignin–phenylpropane and wax. The C2 peak, which corresponds to C–OH/C–O–C mainly from cellulose in bamboo, increased significantly from 14% to 40%. The area of C2 and C3 peaks together signify the presence of oxygenated carbon C_ox_; the area of C1 peak signifies the presence of unoxygenated carbon C_unox_. Accordingly, the ratio of C_ox_/C_unox_ can be calculated from the following Eq. (1)^42^:

1 $${{{{{{\rm{C}}}}}}}_{{{{{{\rm{ox}}}}}}}/{{{{{{\rm{C}}}}}}}_{{{{{{\rm{unox}}}}}}}=\frac{C2+C3}{C1},$$

The C_ox_/C_unox_ ratio of the bamboo materials in Table 1 increased from 0.19 to 0.75 during the delignification process, further indicative of significant oxidation and removal of lignin and hemicellulose (Supplementary Fig. 7 and Tables 1 and 2). Moreover, the 25.3% increase in the C2 peak (C–OH/CO–C) is an indication of more hydrogen bond interactions^14^, which is likely to be advantageous for mechanical performance. The elemental O/C ratio in delignified bamboo, shown in Table 1, was found to be close to that of cellulose^44^. The Ti *2p* peaks in Fig. 1h in fact possess two characteristic peaks of Ti *2p*_*1/2*_ at 464.6 eV and Ti *2p*_*3/2*_ at 458.9 eV in Fig. 2m. The binding energy difference of 5.7 eV between the Ti *2p*_*1/2*_ peak and Ti *2p*_*3/2*_ peak reveals a valence state of +4 for Ti, indicating that Ti was in the form of TiO_2_ in the samples^28^.

**Table 2:** Flexural properties of different bamboo specimens

Materials	Stress [MPa]	Modulus [GPa]	Breaking energy [MJ m^−3^]
Natural	124.5 ± 17.67	10.25 ± 1.63	7.66 ± 1.16
Alkali-Press	332.59 ± 15.39	20.78 ± 2.9	20.08 ± 1.98
TiO_2_-Reinforced	417.91 ± 36.68	31.12 ± 3.79	26.12 ± 3.56

Taken together, the TiO_2_-reinforced densified bamboo was seen to be successfully prepared from natural bamboo, with the anisotropic morphology of cellulose fibers being maintained despite the change in chemical composition.

### Flexural mechanical performance of TiO_2_-reinforced densified bamboo

**Fig. 3:**
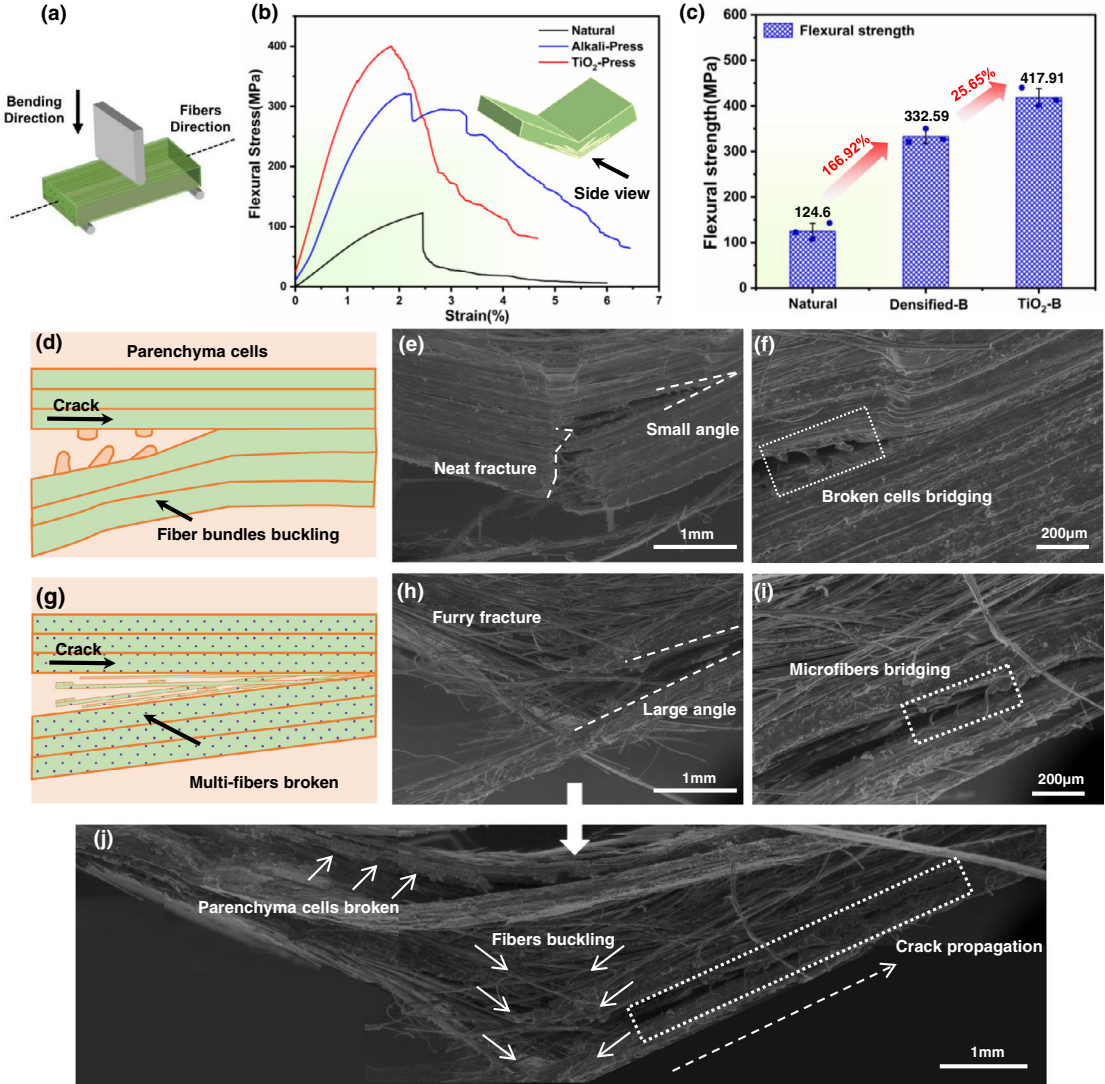
Flexural properties and SEM of the morphology fracture of bamboo. **a** Schematic diagram of the flexural test. **b** Flexural stress-strain curves of natural, densified and TiO_2_-densified bamboo. **c** The improvement of natural, densified and TiO_2_-densified bamboo. Data are presented as mean values ± SEM, *n* = 3 independent samples. **d** Schematic diagram of the morphology of the fracture of densified bamboo. **e**, **f** SEM image of the fracture of densified bamboo, (**e**) neat fracture, (**f**) broken cells bridging. **g** Schematic diagram of the fracture morphology of TiO_2_-densified bamboo. **h**–**j** SEM image of the fracture of TiO_2_-densified bamboo, the furry fracture of (**h**), (**i**), and the full fracture image in (**j**).

The flexural mechanical properties of natural bamboo, densified bamboo, and TiO_2_-reinforced densified bamboo are compared in Fig. 3 and Table 2. The direction of the bending load was the same as the gradient of the fiber density in bamboo. As shown in Fig. 3b, c, alkali-treated densified bamboo showed a flexural strength of 333 MPa, whereas the TiO_2_-reinforced densified bamboo demonstrated the highest flexural strength of 418 MPa, i.e., over 190% larger than that of natural bamboo. The flexural strength of 333 MPa for densified bamboo was similar to that reported in ref. ^27^; after decoration with the TiO_2_ nanoparticles, the flexural strength became higher than the reported 404 MPa for similar densified outer bamboo. Thus, the reinforcement effect of TiO_2_ nanoparticles was clearly significant. The flexural strength of the bamboo material was improved by 26% after TiO_2_ reinforcement, with the flexural modulus increasing from 21 GPa to 31 GPa; additionally, a higher specific flexural strength of 311 MPa cm^3^ g^−1^ was also seen (Supplementary Fig. 8). Moreover, after TiO_2_ reinforcement, the tensile strength increased to 392 MPa*,* i.e., a ~22% improvement (Supplementary Fig. 9). Given that the mass increase from the introduction of TiO_2_ particles was minimal, the effect of reinforcement with 3.8% Ti atoms on the mechanical properties of bamboo can be considered to be particularly significant (Table 1).

The side view of various bamboo materials based on SEM imaging is shown in Fig. 3e–j. It can be seen that the crack path of the densified bamboo was serrated, exhibiting a small deflection angle along the vertical direction under bending (Fig. 3e). Specifically, the hot-pressing procedure compressed the microfiber bundles and parenchyma cells to result in a “sandwich-type” morphology. Nonetheless, cracks tended to propagate along the interface between the cells and fibers leading to delamination (laminar debonding), although crack bridging by the parenchyma cells was also evident (Fig. 3f). However, the overall fracture morphology of the TiO_2_-reinforced densified bamboo was much rougher. A clear fracture feature was the fibrillation of microfiber bundles with the pull-out of multiple bamboo microfibers along with the interfacial delamination of the parenchyma cells (Fig. 3h–j). The vertical crack path in the TiO_2_-reinforced densified bamboo was largely deflected causing the separation of the fibers and the cells (Fig. 3h). The majority of fibers could be seen to be bridging the cracks, which is a potent extrinsic toughening mechanism^34^ that further consumes energy during the fracture of TiO_2_-densified bamboo. For densified bamboo, the delamination of cells and the fracture of microfiber bundles were the two dominant damage modes. In contrast, fibrillation and the fracture of individual fibers were the two dominant damage modes of the TiO_2_-densified bamboo. Supplementary Fig. 10 and 11 also evidence the strengthening enhancement of TiO_2_-densified bamboo; however, it should be noted that although the TiO_2_ nanoparticles were difficult to detect in the middle of the specimens, the occurrence of fibrillation and single fibers buckling still showed a significant influence of TiO_2_ decoration on the bamboo material.

**Fig. 4:**
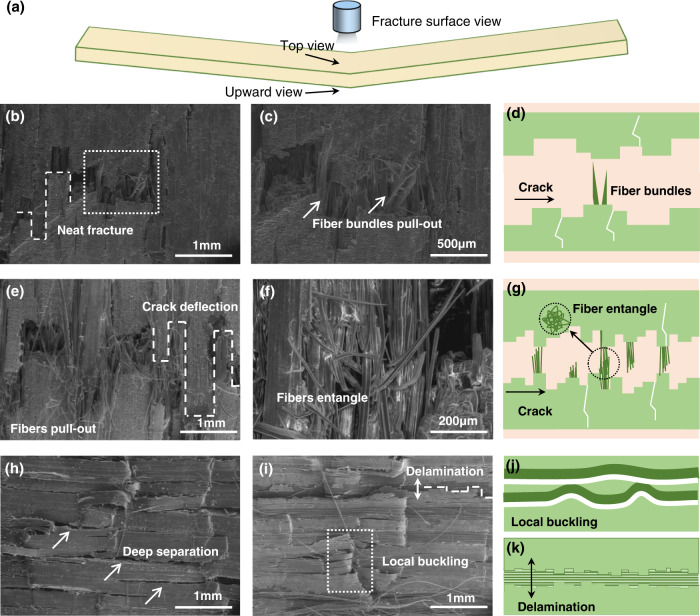
SEM morphology fracture of top and upward view of densified and TiO_2_-densified bamboo. **a** Schematic of the observation view. **b**, **c** Upward view of densified bamboo fracture, (**b**) neat fracture, and the magnification image in (**c**). **d** Schematic diagram of morphology upward view fracture of densified bamboo. **e**, **f** Upward view of TiO_2_-densified bamboo fracture, (**e**) microfibers pulled out, (**f**) fibers entangle. **g** Schematic diagram of morphology upward view fracture of TiO_2_-densified bamboo. **h** Top view SEM morphology of densified bamboo. **i** Top view SEM morphology of TiO_2_-densified bamboo. **j**, **k** Schematic diagram of top view fracture behavior of bamboo, (**j**) local buckling, (**k**) delamination.

Figure 4 shows the morphology of flexural fracture of the bottom and top planes. For the bottom plane with the largest flexural strain, the densified bamboo fracture showed a serrated crack with the pull-out of microfiber bundles (Fig. 4b, c), whereas the fracture of the TiO_2_-reinforced densified bamboo involved a critical fracture mechanism of fibrillation^45^, which refers to the dissociation of microfibrils within the fibers. After fibrillation, the bamboo microfibers were further pulled out with some creating bridges across the serrated crack (Fig. 4e). It is readily apparent that the presence of the TiO_2_ nanoparticles promotes the fibrillation of the microfiber bundles in densified bamboo, which is an important mechanism to enhance the toughness and damage-tolerance of bamboo^46^. Indeed, Fig. 4h, i shows that deep cracks appeared on the top surface of both bamboo materials, indicating delamination and buckling of the fibers and parenchyma cells. Such a buckled or wavy crack morphology can increase the bending stiffness, and the deflection of cracks can further provide extrinsic toughening^47^.

To summarize, the SEM fractography of the bamboo materials revealed that the introduction of 3.8% TiO_2_ nanoparticles resulted in a fracture mode transition. The fracture mode changed from cracking with the pull-out of microfiber bundles to cracking associated with fibrillation and pull-out of bamboo microfibers. The latter mechanisms resulted in a marked increase in the strength and toughness of the bamboo due to the presence of the TiO_2_ reinforcement, quantitatively to a respective improvement in both flexural strength and modulus by ~26% and ~50% (Table 2).

### Real-time flexural fracture process analysis by acoustic emission

**Fig. 5:**
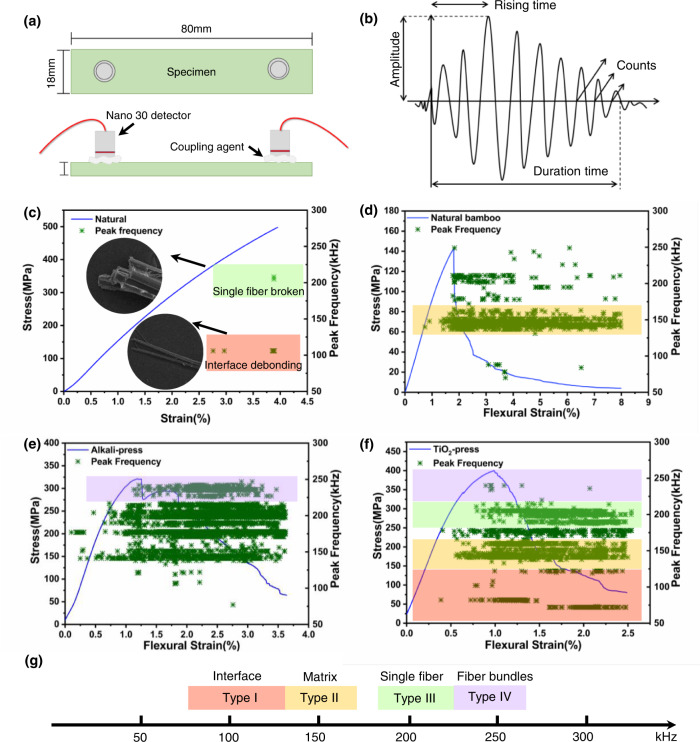
Representative damage modes and real-time acoustic emission (AE) peak frequency signals in natural, densified, and TiO_2_-densified bamboo. **a** Schematic diagram of AE set-up. **b** AE signal characteristics. **c** AE signals of tensile fracture of single bamboo fiber with the identified Type I and Type III signals in red and green highlight. **d** AE signals of natural bamboo flexural process with the identified Type II signals in yellow highlight. **e** AE signals of densified bamboo flexural process with the identified Type IV signals in purple. **f** AE signals of TiO_2_ densified bamboo flexural process with classification of four types of signals. **g** Classification of AE signals. To clarify the signal classification, all types of signals are only highlighted in (**f**).

As noted above, acoustic emission (AE) is a widely used nondestructive analytical tool to monitor the fracture process as well as to distinguish different fracture modes of structural materials (such as matrix fracture, delamination, fiber fracture, etc.)^38,48^. As shown in Fig. 5, AE was employed to monitor the flexural fracture process of two bamboo materials. The AE peak frequency signals can be generally associated with matrix fracture, delamination, and fiber fracture of bamboo at various low to high frequencies. The characteristic AE peak frequency signals of the microfiber bundle fracture and fiber separation / delamination were first identified by stretching a bamboo microfiber bundle (diameter ~90 μm) (Fig. 5c). The corresponding AE signals were concentrated at frequency bands of ~100 kHz for fiber separation / delamination and ~200 kHz for microfiber bundle fracture according to earlier studies^37,38^. Figure 5d–f shows typical AE signals from natural, densified and TiO_2_-reinforced densified bamboo materials during flexural deformation. Due to the unique gradient structure of natural bamboo, a large number of matrix fracture signals were generated and usually concentrated in the frequency band 130–160 kHz^49^ (termed Types I to III). In addition, a large number of 220–250 kHz (Type IV) high-frequency signals was also generated for the densified bamboo (Fig. 5e), which were attributed to microfiber bundle fracture. The frequency signals can thus be classified in terms of 50–130 kHz (Type I), 130–160 kHz (Type II), 180–220 kHz (Type III), and 220–250 kHz (Type IV) in Fig. 5g.

It was clear that the number of Type I acoustic signals from the TiO_2_-reinforced densified bamboo was much higher (Fig. 5f) than that from densified bamboo (Fig. 5e) due to the significantly increased fibrillation and delamination between fibers and TiO_2_-reinforced parenchyma cells (Figs. 3j and 4e); additionally, there was increased friction between the rougher crack surfaces in the TiO_2_-reinforced material (Supplementary Fig. 12). Notably, Type IV signals were significantly less frequent than Type III signals, suggesting that single fiber fracture was more prominent that microfiber bundle fracture.

**Fig. 6:**
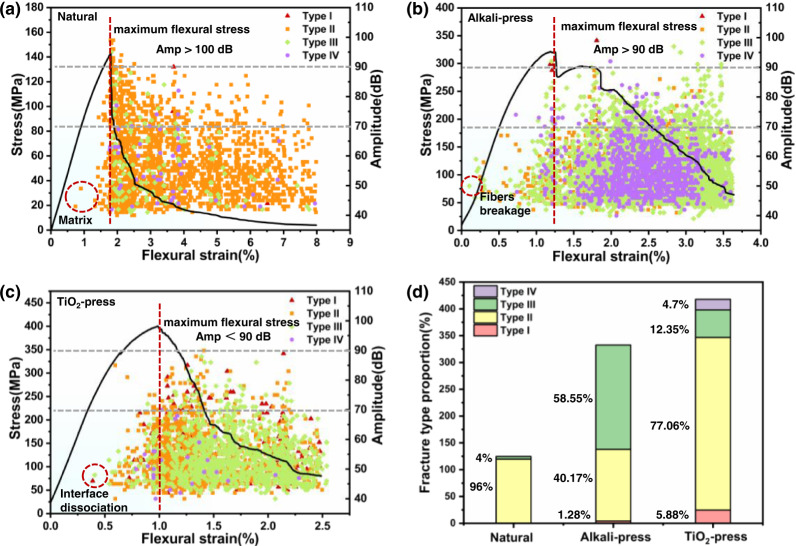
Signal distribution (amplitude and peak frequency) during the flexural deformation of natural, densified, and TiO_2_-densified bamboo. **a** Natural bamboo. **b** Densified bamboo. **c** TiO_2_-densified bamboo. The gray dashed line in (**a**–**c**) divides the amplitude of AE signals into three parts: 30–70, 70–90, and 90–110 dB, respectively. **d** Proportion of different AE signals representing different fracture mechanisms in the three bamboo materials prior to the point of maximum flexural stress (Red dashed line in **a**–**c**).

To understand the acoustic spectra, the amplitude of the AE peaks can be associated with the energy to create the damage. For the various bamboo materials subjected to flexural deformation, the AE amplitude was analyzed to indicate four types of damage modes in Fig. 6. The lower amplitude signals from TiO_2_-densified bamboo indicated more microscale damage with a lower AE energy (shown in Supplementary Fig. 13). This can be related to the fibrillation and friction between fibers and densified parenchyma cells of TiO_2_ densified bamboo. After the maximum flexural stress, ~90% of AE signals were detected. Type II signals characteristic of the matrix cracking were generated, principally in natural bamboo (Fig. 6a), followed by a small number of Type IV signals characteristic of microfiber bundle fracture^50^. In contrast, the densified bamboo generated approximately six times more numerous Type III and Type IV signals than natural bamboo (Fig. 6b). Type I, Type II and Type III signals were dominant for the TiO_2_-reinforced densified bamboo. In particular, Type I signals appeared at the early stage of flexural deformation of the TiO_2_-reinforced densified bamboo, indicating that fibrillation was occurring prior to the fiber pull-out and fracture (Fig. 6c). Clearly as shown in Fig. 6d and Table 3, the number of AE signals associated to various damage modes and energies prior to the maximum flexural stress was quite distinct for the three bamboo materials. Matrix cracking, through densified parenchyma cells, was the most prominent (~96%) in natural bamboo, with matrix fracture and fiber fracture prominent in the densified bamboo. The introduction of TiO_2_ definitively suppressed the fracture of microfiber bundles, from ~59 to 12%, transitioning to single fiber fracture and fiber dissociation/fibrillation. It should be noted that the combination of fracture modes induced in the TiO_2_-reinforced densified bamboo is completely consistent with its more than threefold higher fracture energy compared to natural bamboo. TiO_2_-reinforced densified bamboo definitely displays enhanced toughness.

**Table 3:** Number of Type I–IV AE signals and different amplitude of AE signals prior to the maximum flexural stress

Samples	Peak frequency				Amplitude		
	Type I	Type II	Type III	Type IV	35–70	70–90	>90
Natural	0	72	3	0	46	25	0
Alkali-Press	3	94	137	0	318	21	4
TiO_2_-Press	10	131	21	8	233	9	0

### Toughening mechanisms in TiO_2_-reinforced densified bamboo

As natural fiber composite materials, bamboo materials with a fiber-and-parenchyma cell matrix morphology showed extensive delamination / fibrillation, fiber pull-out, microfiber bridging prior to outright fracture. We revealed, using SEM imaging and acoustic emission (AE), that the dominant strengthening and toughening mechanisms during the flexural deformation can vary significantly depending on whether TiO_2_ particles were introduced into the bamboo material. For the TiO_2_-reinforced densified bamboo, fibrillation of the microfiber bundle and microfiber fracture were most prominent, which resulted in enhanced flexural toughness. Additional toughening was likely achieved through the friction between the bamboo microfibers. Due to these fracture processes, the crack path under flexural loading became significantly tortuous, being deflected within the “fiber-parenchyma cells-fiber” structure of TiO_2_-reinforced densified bamboo, a scenario that further served to increase the toughness of this material. It is noted that these fracture events in TiO_2_-reinforced densified bamboo are active at micrometer length scales, which primarily affect the extrinsic toughness of bamboo materials.

**Fig. 7:**
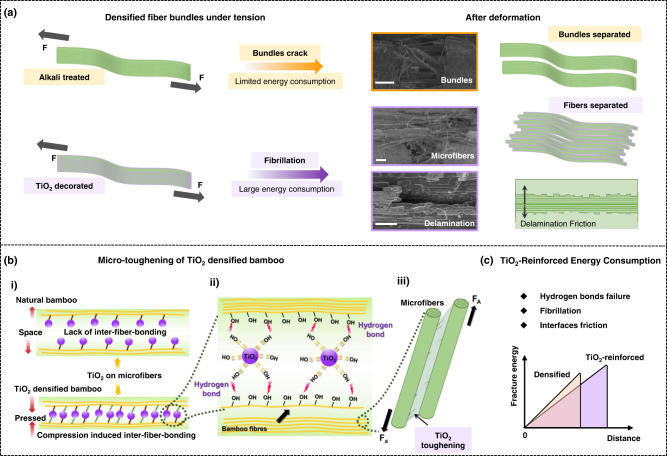
Schematic diagram of the salient mechanisms of how TiO_2_ reinforcements can improve the flexural property of densified bamboo. **a** Deformation to fracture of densified and TiO_2_-reinforced densified bamboo under tension. Scale bars: 200 μm. **b** Micro TiO_2_-reinforcement mechanisms in the flexural deformation process. (i) Bonding mode of TiO_2_ on natural and densified bamboo. (ii) Hydrogen bonds of TiO_2_-reinforced densified bamboo. (iii) TiO_2_ toughening between bamboo microfibers. **c** Energy consumption of TiO_2_-reinforced densified bamboo.

Figure 7 illustrates these fracture events to indicate the specific micro-effects of the TiO_2_ particles in TiO_2_-reinforced densified bamboo. In this material, although the bamboo was compressed and chemically strengthened by the introduction of hydroxyl groups and extensive hydrogen bonding, the interfaces among parenchyma cells remained weakly bonded, leading to cell delamination and microfiber bundle separation and eventually microfiber bundle fracture (Fig. 7a). However, the TiO_2_ particles distributed in the cells and fibers not only exerted a bridging effect between the fibers and matrix, but also rendered the separation of fibers/fibrillation in the microfiber bundles to be more prevalent (as shown in Fig. 7a, b). The angle of the cracks within the fibers also tended to decrease after the addition of TiO_2_ nanoparticles^51^, which may further act to toughen the composite bamboo material by increasing crack deflection and energy dissipation. It is apparent that the fibrillation process in the TiO_2_-reinforced bamboo provides a potent contribution to the flexural toughness of this material.

## Discussion

A three-step processing procedure was applied to transform natural bamboo into a high-flexural-performance bamboo composite. The first step of delignification removed most of the lignin and hemicellulose, introduced a large number of hydroxyl groups, and preserved the cellular morphology and highly oriented fibrous structure of bamboo. The second step of hydrothermal synthesis successfully introduced TiO_2_ nanoparticles in the cells and on the fibers of the bamboo. The final step compressed the material to a compact fiber composite. Such processing led to a significantly elevated flexural strength of 418 MPa for the TiO_2_-reinforced densified bamboo, i.e., three times of that of natural bamboo. We attribute these outstanding flexural properties not only to the introduction of hydrogen bonds between aligned fibers and TiO_2_ nanoparticles but also to microfiber separation, both of which promote energy dissipation during fracture. This study provides a strategy of in situ synthesis of TiO_2_ particles reinforcement for natural porous materials as well as a facile way to identify fracture mechanisms in natural and modified structural materials. The reinforcement of TiO_2_ establishes an efficient means to significantly improve the flexural mechanical performance of natural bamboo, which should expand the structural applications of this promising natural resource.

## Methods

### Materials and chemicals

*Moso* bamboos were purchased from the Hunan province of China and were processed into cuboid specimens with two sets of sizes, 80 × 18 × 6 mm for natural bamboo, and 80 × 18 × 1.5 mm for densified bamboo. Specimens were cut at ~1.5 m from the bottom of 5-year-old mature bamboo. NaOH (AR, Beijing chemical reagent company) and Na_2_SO_3_ (AR, Beijing chemical reagent company) were used in the delignification process to remove lignin and hemicellulose. C_2_H_6_O (AR, Beijing Chemical Reagent Company) and tetrabutyl titanate (≥99.0%, Aladdin) were used to synthesize the TiO_2_.

### Preparation of TiO_2_-decorated bamboo

To explore the effect of synthesis conditions on TiO_2_ nanoparticle formation, tetrabutyl titanate (TNBT) with varied concentrations, 0.015 M, 0.025 M, and 0.035 M, was dissolved in ethanol absolute, respectively, and the solutions stirred for 30 min; the bamboo was then submerged in the solution under a low vacuum (0.1 bar) for 2 h. Subsequently, the bamboo specimens that were impregnated with the TNBT solution were put into a reaction bath at 110 °C for 4 h. After ultrasonic cleaning in ethanol absolute and deionized water for multiple times, the specimens were dried in an oven at 60 °C for further SEM and flexural mechanical tests (Supplementary Figs. 14 and 15). Owing to its appropriate particle size and distribution, 0.025 M TNBT was chosen as the solution for the investigation of the role of TiO_2_ nanoparticle reinforcements in influencing the flexural fracture behavior of densified bamboo.

### Preparation of densified-TiO_2_ bamboo

The bamboo specimens were manufactured into densified TiO_2_ bamboo using three steps. In the first step, bamboo specimens were boiled in 2.5 M NaOH and 0.4 M Na_2_SO_3_ for 8 h, then the specimens were washed in water several times to remove residual alkali until the pH reached ~7. Then 5 mL tetrabutyl titanate was dissolved in 200 mL of absolute ethyl alcohol and the solution stirred for 30 mins; the delignified bamboo was then submerged in the solution under a low vacuum (0.1 bar) for 2 h. Subsequently, the delignified bamboo specimens that were impregnated with solution were put into a reaction bath at 110 °C for 4 h. The processed specimens were pressed at 120 °C under a compressive stress of 6.5 MPa for 12 h to generate the delignified, TiO_2_-reinforced and densified bamboo. The raw bamboo without any treatment, delignified bamboo followed by hot pressing, and TiO_2_- decorated densified bamboo were termed as “natural bamboo”, “densified bamboo” and “TiO_2_-densified bamboo”, respectively. The lignin content (Klason lignin) of these bamboo materials was measured according to the TAPPI method (TAPPI T 222 om-02)^52^: Specifically, 3 mL 72 wt.% H_2_SO_4_ was added into 0.2 g (*M*) bamboo powder and reacted for 1 h. The H_2_SO_4_ solution was then diluted to 3 wt.% and the mixture heated in an oven at 120 °C ± 3 °C for 1 h. After the solution was cooled down and filtered, the residual was dried at 105 °C ± 3 °C until the mass (*m*) was stable. The content of lignin was calculated according to Eq. (2):

2 $${{{{{\rm{Content}}}}}}\; {{{{{\rm{of}}}}}}\; {{{{{\rm{lignin}}}}}}=\frac{m}{M}\times 100\%$$

The density of our bamboo specimens was measured as follows. All the specimens were dried in an air-flow oven at 80 °C ± 3 °C until the mass of the specimens was stable. The specimens were then placed under the same ambient conditions (20–25 °C, 30–40% relative humidity) to reach the same hydration state, prior to the mass *M* being measured. The volume of the specimens did not change during such ambient conditioning; their size also did not change after the treatment. Cuboid specimens with the above-mentioned sizes, corresponding to volumes of *V* = 8640 mm^3^ for natural bamboo and *V* = 2160 mm^3^ for densified bamboo. The density was calculated from *M*/*V*, based on measurements on three specimens; mean values are presented.

### Mechanical testing

The tensile and flexural tests of bamboo specimens were conducted on a mechanical testing machine (SANS, MTS Industrial System Co. Ltd., China) operating at a displacement rate of 1 mm/min under ambient conditions. The dimensions of these specimens were 80 × 18 × 6 mm for natural bamboo and 80 × 18 × 1.5 mm for densified and decorated densified bamboo, respectively; all samples were unnotched. Strain-stress curves were generated using origin software (orignlab 9.0), and illustrations were finalized using Power Point 2013 software.

### Microstructure and morphology analysis

Fourier transform infrared (FTIR) spectroscopy of the specimens was determined using a Nicolet Magna IR560, spectrometer, with the spectral range from 4000 to 400 cm^−1^. A field-emission scanning electron microscope (FESEM, ZEISS ULTRA 55, Germany) with an accelerating voltage of 10 kV and a low vacuum mode was used to characterize the morphologies of natural and densified-TiO_2_ bamboo using the secondary electron mode. The chemical characterization of Ti was analyzed in an energy-dispersive spectrometer (EDS) equipped with the FESEM. Computed micro-tomography was performed at beamline 4W1A at the Beijing Synchrotron Radiation Facility (BSRF, Beijing, China) with an X-ray energy of 15 keV. Fracture morphologies were observed using an optical microscope (OM, Leica DM400, Leica Microsystems, Germany). The modified, as well as the unmodified, bamboo was analyzed using X-ray diffraction (XRD, Rigaku Corp., Tokyo, Japan) by Jade software v6.2. X-ray photoelectron spectroscopy (XPS) was measured (ULVAC–PHI Quantera SXM, INC) at a pass energy of 0.1 eV with Al Kα as the X-ray source, and the data was analyzed by XPSpeak software v4.1. Small-angle X-ray scattering (SAXS) was performed at 1W2A beamline of the Beijing Synchrotron Radiation Facility using a beam size of 1 × 0.4 mm^2^, with the 2D scattering patterns collected from the data processing software Fit2D (v18.002).

### Acoustic emission

The acoustic emission (AE) technique was applied to detect the acoustic signals during flexural mechanical test arising from damage and cracking events. All the signals were collected by two piezoelectric sensors (Nano-30, frequency of 140 kHz with band-pass filtering of 150–400 kHz), and were analyzed using a digital signal processor with an AEwin v3.61 AE system (Physical Acoustic Corporation, USA). To improve the conduction of the signals between the specimens and detectors, Vaseline was smeared on the surface of sensors. To filter the noise acoustic signals from other environmental sources, an amplitude acquisition threshold of 40 dB was used in all experiments.

### Reporting summary

Further information on research design is available in the Nature Portfolio Reporting Summary linked to this article.

## Supplementary information

Supplementary Information

Reporting Summary

## Data Availability

The data that support the findings of this study are available within this paper or included in the Supplementary Information. Source data are provided with this paper.

## Source data

Source Data
