# Supplementary material for: Robust flexural performance and fracture behavior of TiO2 decorated densified bamboo as sustainable structural materials
Source: Nat Commun. 2023 Mar 4;14:1234. doi: 10.1038/s41467-023-36939-6 (PMC9985615; doi:10.1038/s41467-023-36939-6)
Supplement: Supplementary file 1 — Supplementary Information [file 41467_2023_36939_MOESM1_ESM.pdf]

Supplementary Information

Robust Flexural Performance and Fracture Behavior of TiO_2 Decorated Densified Bamboo as Sustainable Structural Materials

Ziyu Ba,[1] Hongyun Luo,[1,2,3*] Juan Guan,[1,2] Jun Luo,[1] Jiajia Gao,[1]

Sujun Wu,[1] Robert O. Ritchie[4*]

[1] School of Materials Science and Engineering, Beihang University, Beijing, P. R. China.

[2] Beijing Advanced Innovation Centre for Biomedical Engineering, Beihang University, Beijing, P. R. China.

[3] Beijing Key Laboratory of Advanced Nuclear Materials and Physics, Beihang University, Beijing, P. R. China.

[4] Department of Materials Science & Engineering, University of California, Berkeley, CA 94720, USA.

*Email: roritchie@lbl.gov (R.O.R); Luo7128@163.com (HY.L)

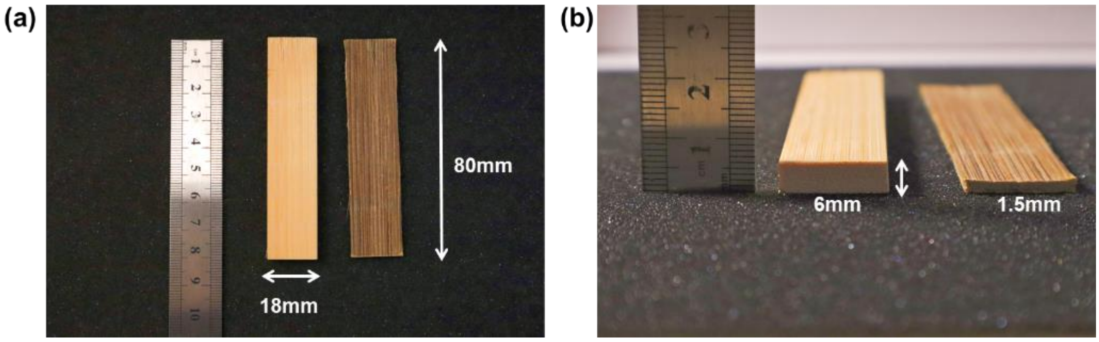

Supplementary Figure 1 | Images of natural and TiO_2 densified bamboo. a Top view of natural and TiO_2 densified bamboo. **b** Lateral view of natural and TiO_2 densified bamboo.

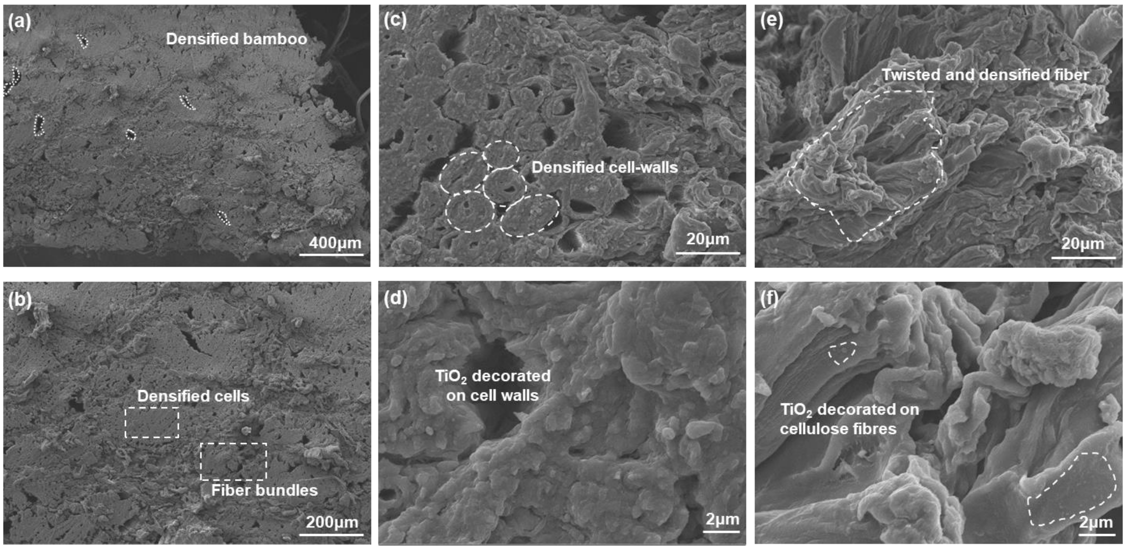

Supplementary Figure 2 | SEM images of the morphology of parenchyma cells and fibers in TiO_2-reinforced densified bamboo. a-b The overall morphology showing significantly compressed parenchyma cells. **c-d** Parenchyma cells with TiO_2. **e-f** Twisted and compressed cellulose fibers.

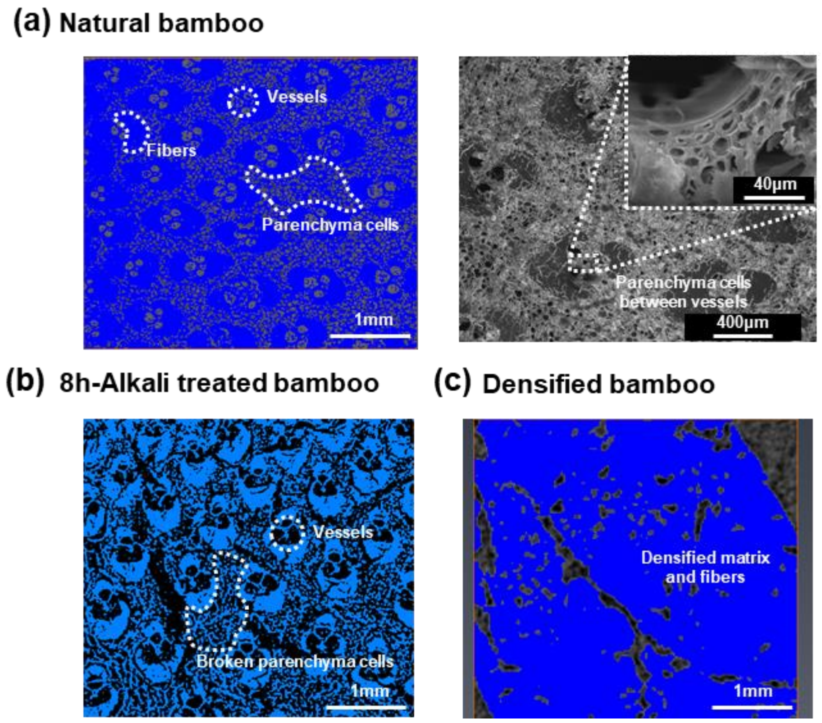

Supplementary Figure 3 | Micro-computed tomography of various of bamboo materials. **a** Natural bamboo. **b** Delignified bamboo after the 8-h alkali treatment. **c** Densified bamboo. In natural bamboo, the diameter of the vessels is ~111 to 143 μm, and the diameter of the parenchyma cells is ~52 to 78 μm. After the delignification process, the parenchyma cells between vessels were broken, so the diameter of the voids between vessels increased to ~190 μm, nearly doubled the size of pores in natural bamboo.

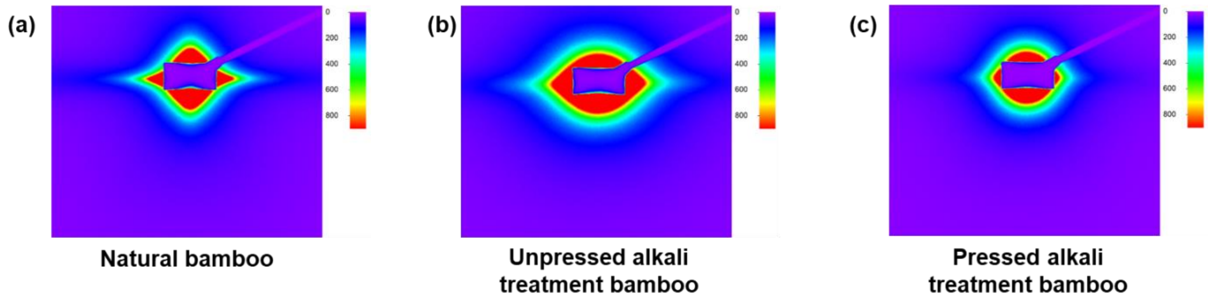

Supplementary Figure 4 | Small-angle X-ray scattering (SAXS) patterns of natural bamboo, alkali treated bamboo without compression and densified bamboo. a Natural bamboo. **b** Uncompressed delignified bamboo. **c** Densified bamboo.

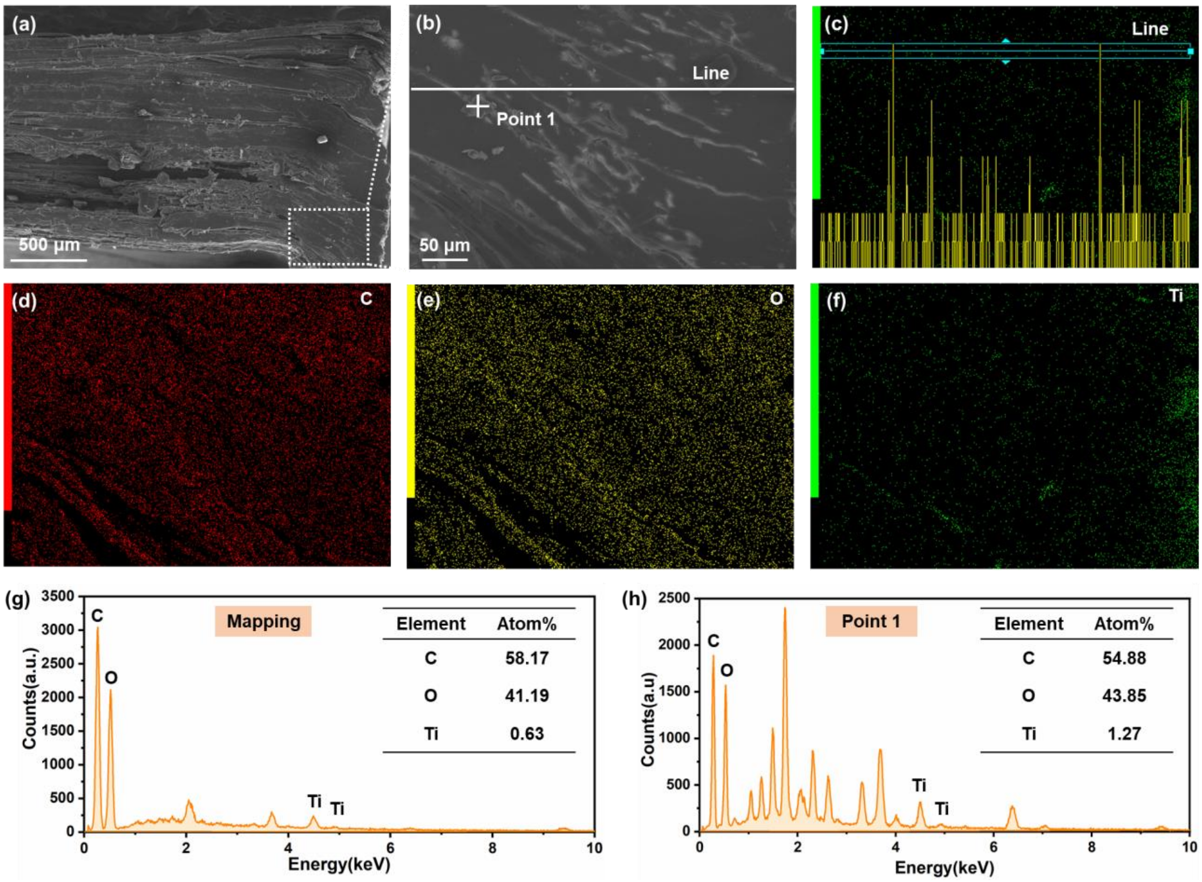

Supplementary Figure 5 | SEM and EDS images of lateral section of TiO_2 densified bamboo. a Lateral section of TiO_2 densified bamboo. **b** Position for EDS line and point scanning mode. **c** Line scanning for Titanium, yellow line shows the element intensity**. d-f** Relative chemical element distribution and atomic ratio of C, O, Ti, detected by EDS in the magnified region. Red: Carbon, yellow: Oxygen, green: Titanium. **g** Elements distribution and atomic ratio of map scanning of (**b**). **h** Elements distribution and atomic ratio of point scanning in (**b**).

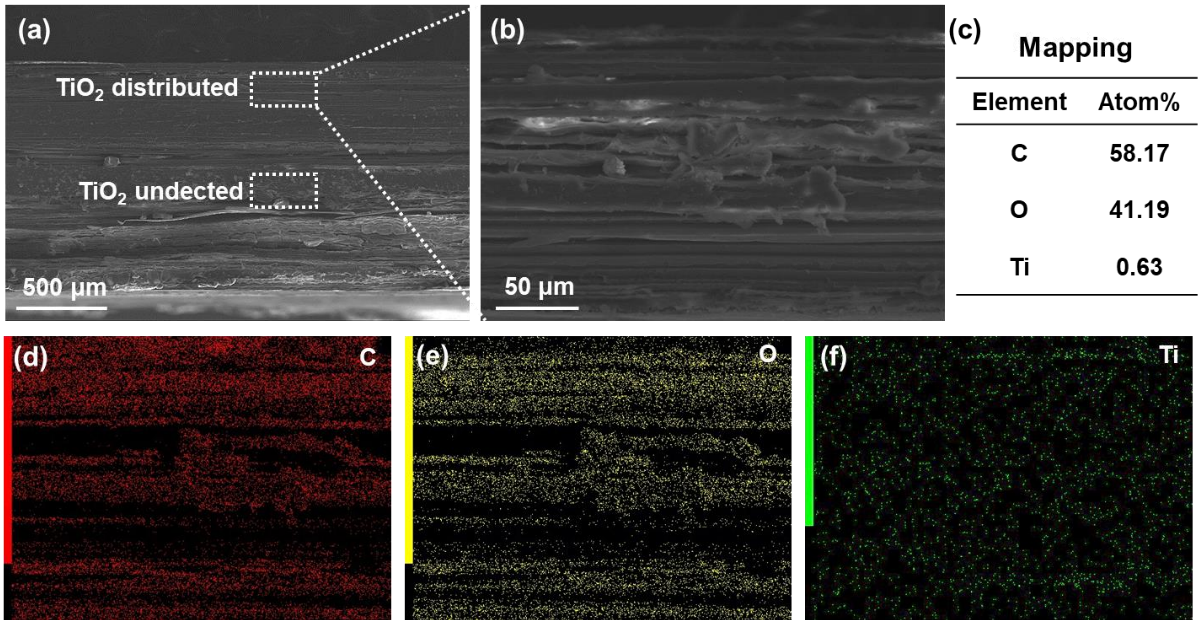

Supplementary Figure 6 | SEM and EDS images of the middle of the lateral section of TiO_2 densified bamboo. a SEM morphology of lateral section of TiO_2 densified bamboo. **b** EDS mapping area. **c** The atomic ratio of C,O and Ti of EDS mapping. **d-f** Element distribution of C, O and Ti. Red: Carbon, yellow: Oxygen, green: Titanium. The thickness of TiO_2 densified bamboo was 1.2 to 1.5 mm; TiO_2 can be detected in the region ~0.5 mm from the surface. In the middle of the densified bamboo (~0.75 mm to the surface), the TiO_2 nanoparticles were barely detected, which suggests that the reaction solution could not infiltrate to that depth where TiO_2 particle synthesis was not initiated.

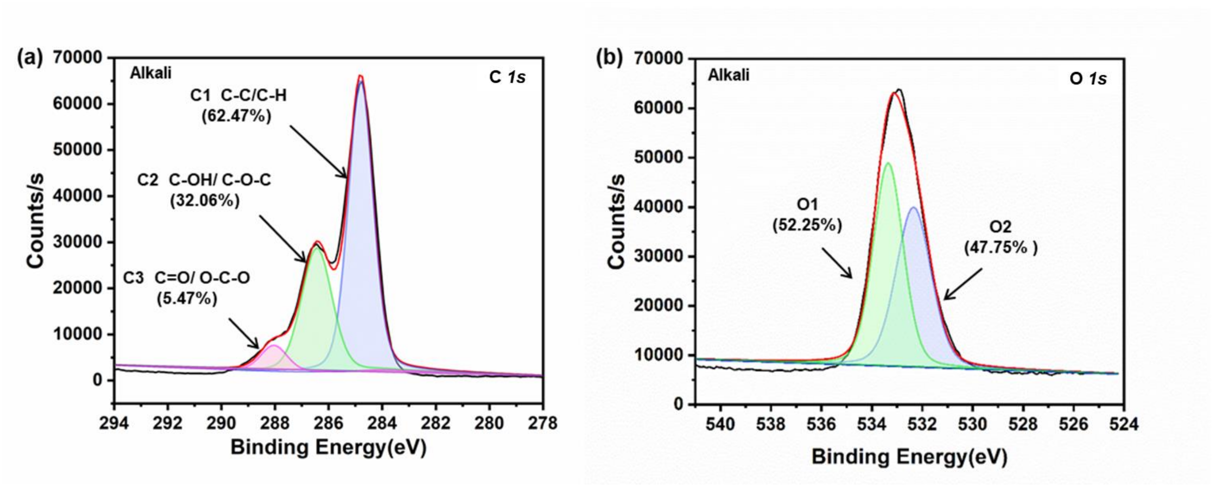

Supplementary Figure 7 | High-resolution XPS spectra of densified bamboo. a C *1s* peaks of densified bamboo. **b** O *1s* peaks of densified bamboo.

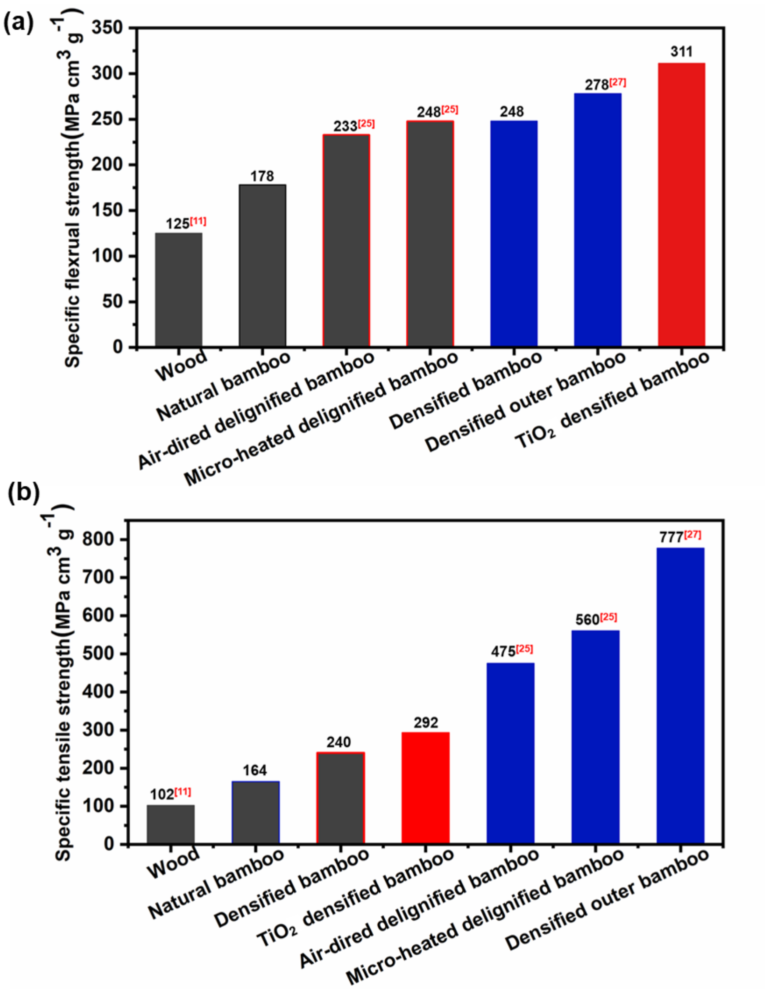

Supplementary Figure 8 | Specific strength of different bamboo materials. a Specific flexural strength. **b** Specific tensile strength. TiO_2 densified bamboo showed competitive specific flexural strength due to the reinforcement of nanoparticles in densified bamboo. The property data of the composites are taken from this work and the references. Reference numbers corresponding to the main document are noted above each column, and the specific value is also noted.

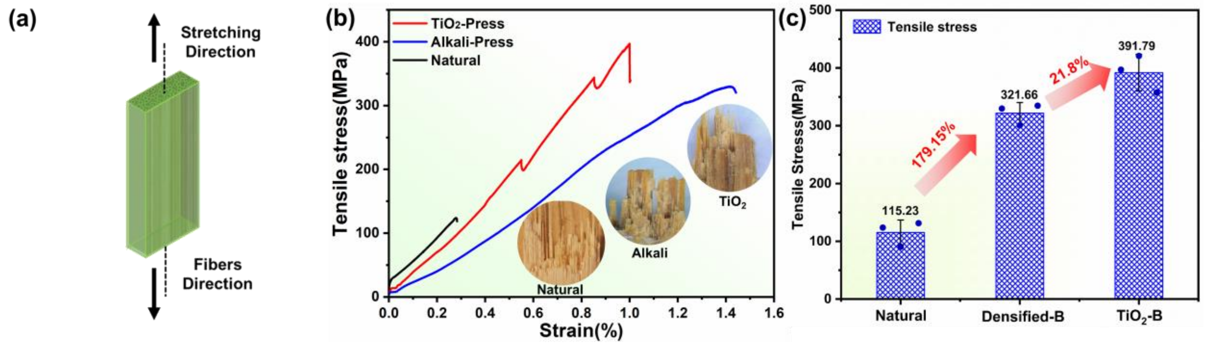

Supplementary Figure 9 | Tensile strength of natural, densified and TiO_2-reinforced bamboo. a Schematic diagram of the uniaxial tensile test. **b** Tensile stress-strain curves of natural, densified and TiO_2-densified bamboo. **c** The improvement in the tensile strength of densified and TiO_2-densified bamboos as compared to natural bamboo. Data are presented as mean values ± SEM, n = 3 independent samples.

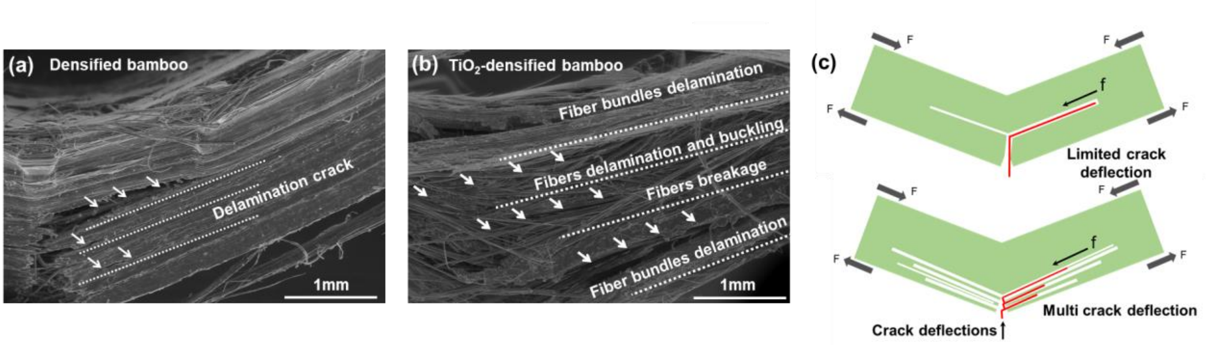

Supplementary Figure 10 | Flexural morphology of bamboo materials at the micrometer scale with illustration of the crack deflection. a Delamination or separation of fiber bundles in densified bamboo; **b** Separation of fiber bundles and fibers and fiber breakage in TiO_2-densified bamboo. **c** Schematic illustration of crack deflection during flexural deformation of densified bamboo and TiO_2-reinforced densified bamboo;

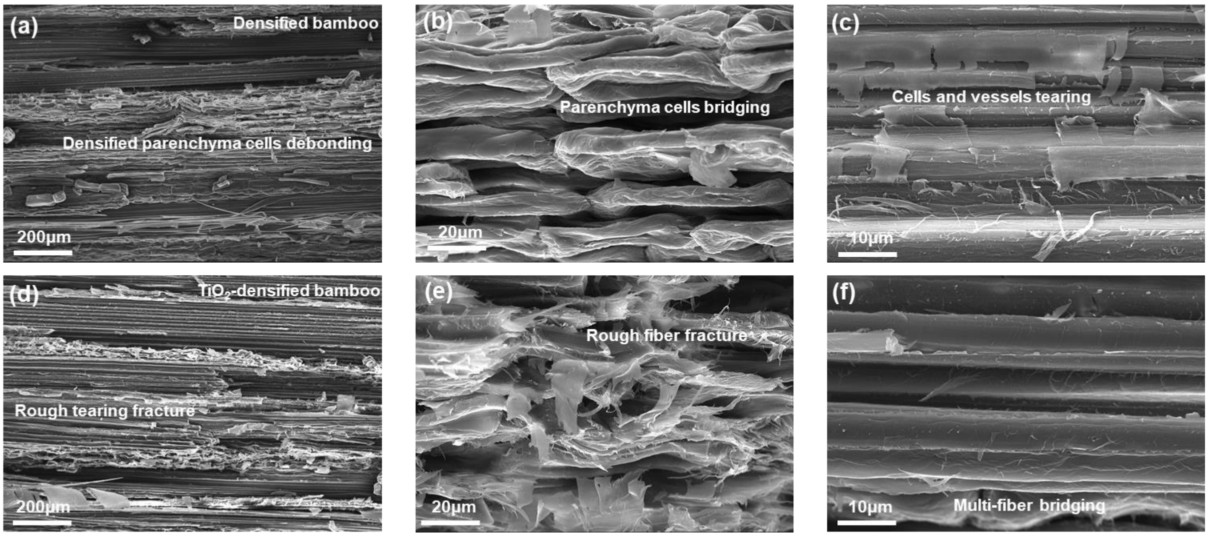

Supplementary Figure 11 | Longitudinal morphology of the fracture: of **a-c** densified and **d-f** TiO_2-reinforced densified bamboo. Parenchyma cell debonding and bridging, cells and vessels tearing are shown in a-c for densified bamboo. Rougher debonding and tearing fracture, fiber fracture and bridging are shown in d-f for TiO_2-reinforced densified bamboo.

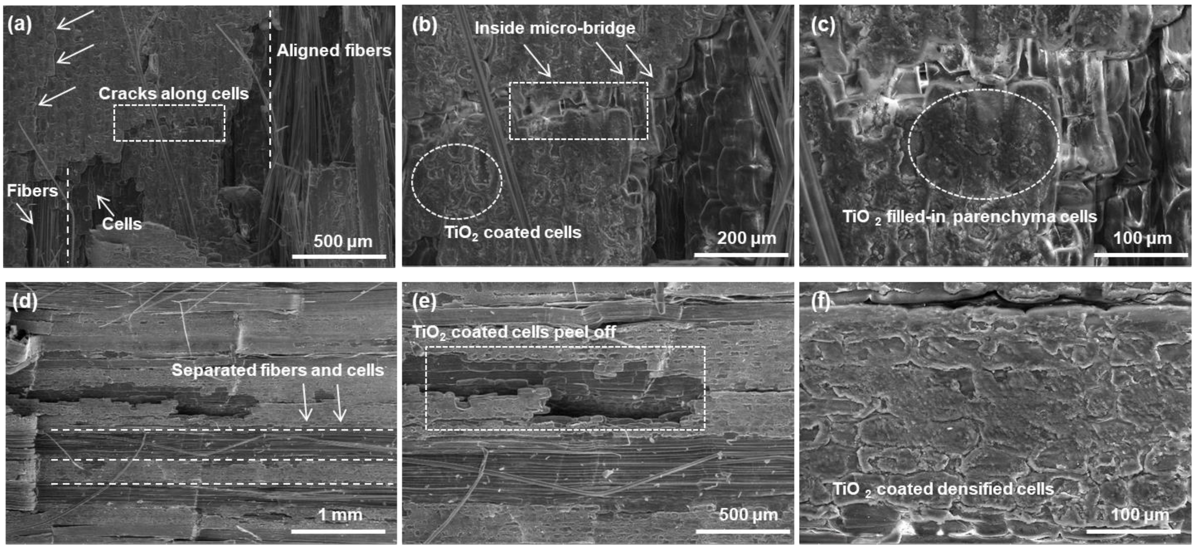

Supplementary Figure 12 | SEM images of the distribution of the TiO_2 in densified bamboo. a-c Upward view. The arrows in the top left corner of **a** indicate the cracks along the interface of fibers and cells; the region in the middle is the broken parenchyma cell as separated by the dotted lines from the fiber region, and the rectangular region shows cracks in the cells. The oval region in **b** shows the TiO_2 coated cells and the rectangular region shows micro-bridges among cells. The oval region in **c** shows TiO_2 filled-in the cells. **d-f** Top view. The dotted lines in **d** indicate the separated fibers and cells. The rectangular region in **e** indicates the cells which peeled-off with decorated TiO_2. All the cells were coated with TiO_2 in **f**.

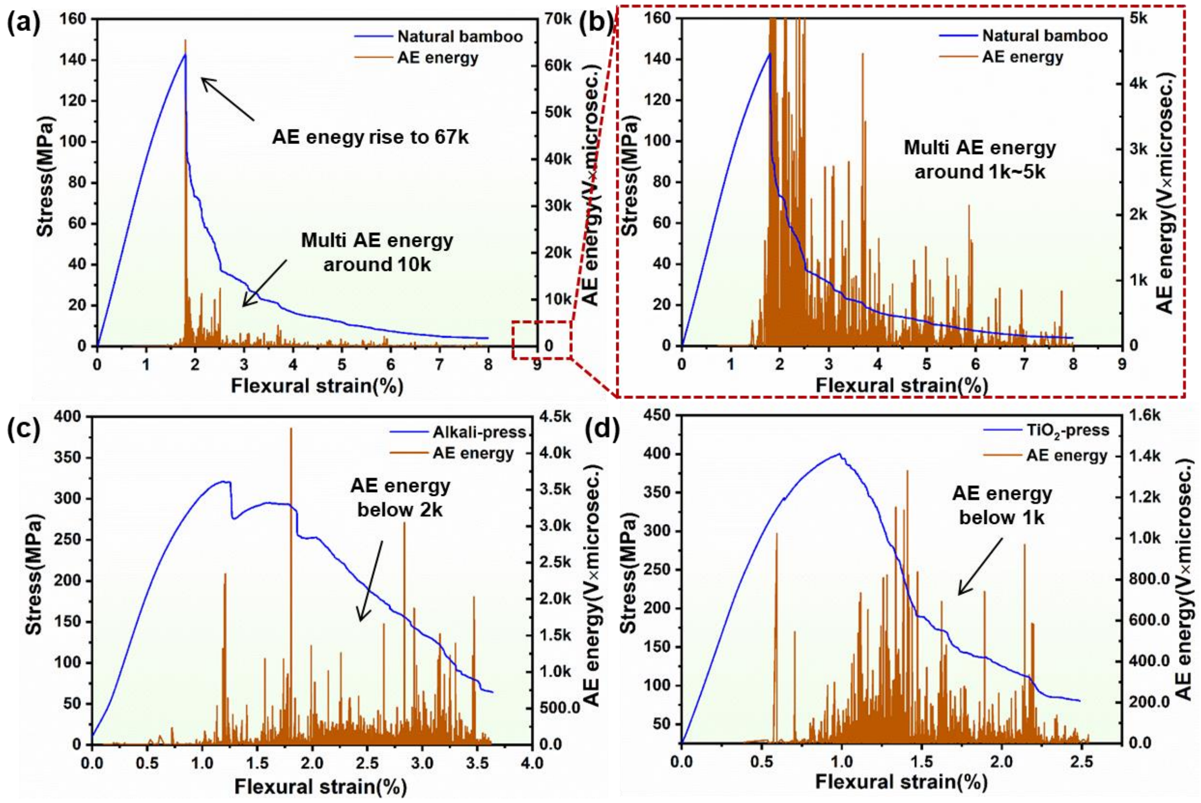

Supplementary Figure 13 | AE energy signals from natural, densified and TiO_2-reinforced bamboo during the flexural deformation. AE energy signals of **a** natural bamboo. **b** natural bamboo below 5k. **c** densified bamboo. **d** TiO_2-reinforced bamboo. During the flexural deformation of natural bamboo, high AE energy signals were generated up to 67k. Additionally, multiple (~1k-5k) AE energy signals were detected, indicating that catastrophic failure occurred in the bamboo material at that moment. In the densified bamboo, however, the AE energy signals were all below 4.5k. In the TiO_2-reinforced bamboo, AE energy signals were much lower (below 1k, and most around 200); this reveals the occurrence of gradual micro-scale damage (fibrillation and microfiber breakage) rather than catastrophic failure.

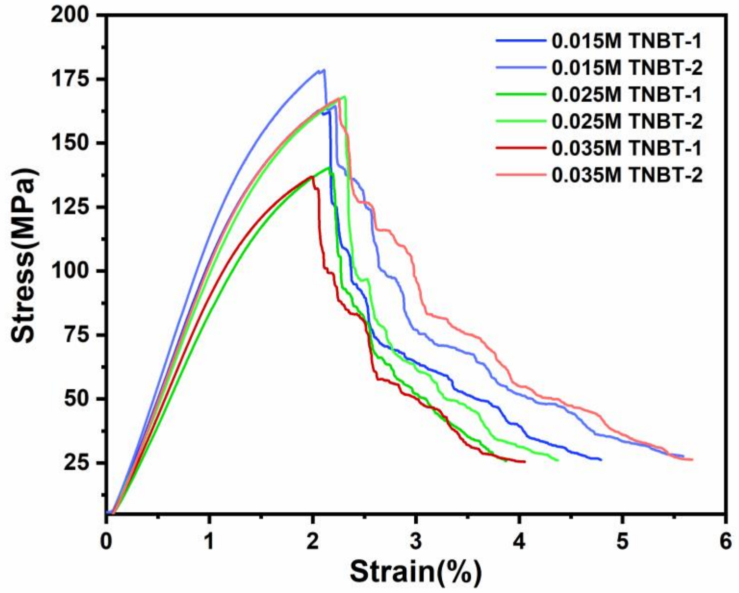

Supplementary Figure 14 | Flexural strength of bamboo with tetrabutyl titanate (TNBT) at different concentrations (from 0.015 M to 0.035 M).

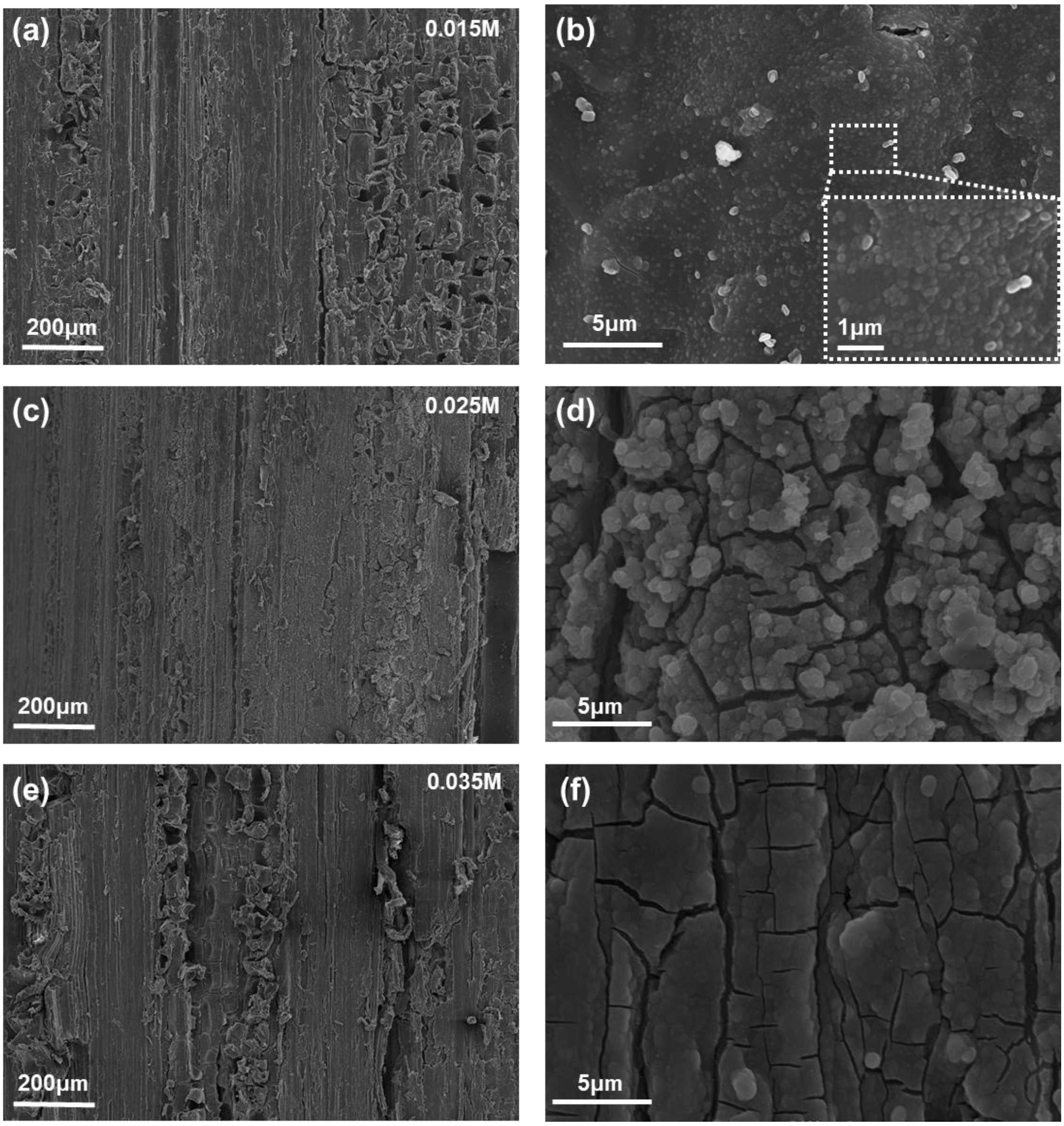

Supplementary Figure 15 | SEM morphology of bamboo with tetrabutyl titanate (TNBT) under different concentrations (from 0.015 M to 0.035 M). a, b TiO_2 nanoparticles synthesized by 0.015 M TNBT; **c, d** TiO_2 nanoparticles synthesized by 0.025 M TNBT; **e, f** TiO_2 nanoparticles synthesized by 0.035 M TNBT. TiO_2 nanoparticles under 0.015 M TNBT displayed a uniform distribution with particle dimensions of ~100 nm. TiO_2 nanoparticles under 0.025 M TNBT showed a much denser distribution; the dimensions of these nanoparticles were ~500 nm to 1 μm. However, the higher concentration of TNBT of 0.035 M did not lead to further improvements in the nanoparticle distribution; in fact, the TiO_2 particles became aggregated and accordingly were hard to be individually seen. Based on the approximate flexural properties of bamboo materials with different TNBT concentration and the resulting morphologies of the synthetic TiO_2 nanoparticles, 0.025 M TNBT was chosen as the medium to fabricate the TiO_2 densified bamboo in this work.

Supplementary Table 1. Experimental atomic composition (%) and O/C ratios obtained by XPS analysis

	**C *1s***	**O *1s***	**Ti**	**O/C**
Natural	81.27	18.73	\	0.23
Alkali treated	72.98	27.02	\	0.37
TiO_2 reinforced	60.3	35.91	3.79	0.6

Supplementary Table 2. Peak deconvolution results of the C *1s* peak (the corresponding bonds) and the oxygenation ratio (C_{ox}/C_{unox})

	**C1**	**C2**	**C3**	**Cox/Cunox**
Natural	83.75%	14.43%	1.82%	0.19
Alkali treated	62.47%	32.06%	5.47%	0.6
TiO_2 reinforced	57.23%	39.73%	3.04%	0.75
