# Supplementary material for: Robust flexural performance and fracture behavior of TiO2 decorated densified bamboo as sustainable structural materials
Source: Nat Commun. 2023 Mar 4;14:1234. doi: 10.1038/s41467-023-36939-6 (PMC9985615; doi:10.1038/s41467-023-36939-6)
Supplement: Supplementary file 2 — Reporting Summary [file 41467_2023_36939_MOESM2_ESM.pdf]

nature portfolio

Corresponding author(s): Robert O. Ritchie and Hongyun Luo

Last updated by author(s): Feb 21, 2023

Reporting Summary

Nature Portfolio wishes to improve the reproducibility of the work that we publish. This form provides structure for consistency and transparency in reporting. For further information on Nature Portfolio policies, see our Editorial Policies and the Editorial Policy Checklist.

Statistics

For all statistical analyses, confirm that the following items are present in the figure legend, table legend, main text, or Methods section.

n/a	Confirmed	
☐	☒	The exact sample size (n) for each experimental group/condition, given as a discrete number and unit of measurement
☐	☒	A statement on whether measurements were taken from distinct samples or whether the same sample was measured repeatedly
☒	☐	The statistical test(s) used AND whether they are one- or two-sided<br>*Only common tests should be described solely by name; describe more complex techniques in the Methods section.*
☒	☐	A description of all covariates tested
☒	☐	A description of any assumptions or corrections, such as tests of normality and adjustment for multiple comparisons
☐	☒	A full description of the statistical parameters including central tendency (e.g. means) or other basic estimates (e.g. regression coefficient) AND variation (e.g. standard deviation) or associated estimates of uncertainty (e.g. confidence intervals)
☒	☐	For null hypothesis testing, the test statistic (e.g. F, t, r) with confidence intervals, effect sizes, degrees of freedom and P value noted<br>*Give P values as exact values whenever suitable.*
☒	☐	For Bayesian analysis, information on the choice of priors and Markov chain Monte Carlo settings
☒	☐	For hierarchical and complex designs, identification of the appropriate level for tests and full reporting of outcomes
☒	☐	Estimates of effect sizes (e.g. Cohen's d, Pearson's r), indicating how they were calculated

Our web collection on statistics for biologists contains articles on many of the points above.

Software and code

Policy information about availability of computer code

Data collection	AEwin v3.61(Physical Acoustics Corporation) was used to collected the acoustic emission signals.
Data analysis	Small-angle X-ray scattering (SAXS) was analyzed by the data processing software Fit2D (v18.002).Strain-stress curves were generated using origin software (orignlab 9.0) . X-ray diffraction was analyzed by Jade software v6.2. X-ray photoelectron spectroscopy was analyzed by XPSpeak software v4.1.The analysis of graphs of SEM and optical microscope used Power Point 2013 software.

For manuscripts utilizing custom algorithms or software that are central to the research but not yet described in published literature, software must be made available to editors and reviewers. We strongly encourage code deposition in a community repository (e.g. GitHub). See the Nature Portfolio guidelines for submitting code & software for further information.

Data

Policy information about availability of data

All manuscripts must include a data availability statement. This statement should provide the following information, where applicable:

- Accession codes, unique identifiers, or web links for publicly available datasets
- A description of any restrictions on data availability
- For clinical datasets or third party data, please ensure that the statement adheres to our policy

The data that support the findings of this study are available within this paper or included in the Supplementary Information. Source data are provided with this paper.

Human research participants

Policy information about studies involving human research participants and Sex and Gender in Research.

Reporting on sex and gender	N/A
Population characteristics	N/A
Recruitment	N/A
Ethics oversight	N/A

Note that full information on the approval of the study protocol must also be provided in the manuscript.

Field-specific reporting

Please select the one below that is the best fit for your research. If you are not sure, read the appropriate sections before making your selection.

☐ Life sciences ☐ Behavioural & social sciences ☒ Ecological, evolutionary & environmental sciences

For a reference copy of the document with all sections, see nature.com/documents/nr-reporting-summary-flat.pdf

Ecological, evolutionary & environmental sciences study design

All studies must disclose on these points even when the disclosure is negative.

Study description	A facile processing, involving delignification, in situ hydrothermal synthesis of TiO2 and pressure densification, is employed to transform natural bamboo into a high-performance structural material.
Research sample	Moso bamboos were purchased from the Hunan province of China and were processed into cuboid specimens with two sets of sizes, 80 mm × 18 mm × 6 mm for natural bamboo, and 80 mm × 18 mm × 1.5 mm for densified bamboo. Specimens were cut at ~1.5 m from the bottom of 5-year-old mature bamboo.
Sampling strategy	N/A
Data collection	The tensile and flexural tests of bamboo specimens were conducted on a mechanical testing machine (SANS, MTS Industrial System Co. Ltd., China) operating at a displacement rate of 1 mm/min under ambient conditions. Strain-stress curves were generated using origin software (orignlab 9.0) . Fourier transform infrared (FTIR) spectroscopy of the specimens was determined using a Nicolet Magna IR560, spectrometer, with the spectral range from 4000 to 400 cm−1. A field-emission scanning electron microscope (FESEM, ZEISS ULTRA 55, Germany) with an accelerating voltage of 10 kV and a low vacuum mode was used to characterize the morphologies of natural and densified-TiO2 bamboo using the secondary electron mode. The chemical characterization of Ti was analyzed in an energy-dispersive spectrometer (EDS) equipped with the FESEM. The analysis of graphs of SEM and optical microscope used Power Point 2013 software. Computed micro-tomography was performed at beamline 4W1A at the Beijing Synchrotron Radiation Facility (BSRF, Beijing, China) with an X-ray energy of 15 keV. Fracture morphologies were observed using an optical microscope (OM, Leica DM400, Leica Microsystems, Germany). The modified, as well as the unmodified, bamboo was analyzed using X-ray diffraction (XRD, Rigaku Corp., Tokyo, Japan). X-ray diffraction was analyzed by Jade software v6.2. X-ray photoelectron spectroscopy (XPS) was measured (ULVAC–PHI Quantera SXM, INC) at a pass energy of 0.1eV with Al Kα as the X-ray source. X-ray photoelectron spectroscopy was analyzed by XPSpeak software v4.1. Small-angle X-ray scattering (SAXS) was performed at 1W2A beamline of the Beijing Synchrotron Radiation Facility using a beam size of 1 × 0.4 mm2, with the 2D scattering patterns collected from the data processing software Fit2D (v18.002). The AE signals were collected by two piezoelectric sensors (Nano-30, frequency of 140 kHz with band-pass filtering of 150-400 kHz), and were analyzed using a digital signal processor with an AEwin v3.61 AE system (Physical Acoustic Corporation, USA).
Timing and spatial scale	All the specimens were under the same timing and spatial scale to acquire the results in experiments.
Data exclusions	No data was excluded.
Reproducibility	Tests were repeated and the results were reproduced in the reported error range.
Randomization	All the bamboo specimens were selected randomly.
Blinding	The study mainly focused on the mechanical properties and mechanisms of specimens. Blinding was not relevant to the study.

Did the study involve field work? ☐ Yes ☒ No

Reporting for specific materials, systems and methods

We require information from authors about some types of materials, experimental systems and methods used in many studies. Here, indicate whether each material, system or method listed is relevant to your study. If you are not sure if a list item applies to your research, read the appropriate section before selecting a response.

Materials & experimental systems

n/a	Involved in the study
☒	☐ Antibodies
☒	☐ Eukaryotic cell lines
☒	☐ Palaeontology and archaeology
☒	☐ Animals and other organisms
☒	☐ Clinical data
☒	☐ Dual use research of concern

Methods

n/a	Involved in the study
☒	☐ ChIP-seq
☒	☐ Flow cytometry
☒	☐ MRI-based neuroimaging
